# 3D VMAT Verification Based on Monte Carlo Log File Simulation with Experimental Feedback from Film Dosimetry

**DOI:** 10.1371/journal.pone.0166767

**Published:** 2016-11-21

**Authors:** A. R. Barbeiro, A. Ureba, J. A. Baeza, R. Linares, M. Perucha, E. Jiménez-Ortega, S. Velázquez, J. C. Mateos, A. Leal

**Affiliations:** 1 Dpto. Fisiología Médica y Biofísica, Universidad de Sevilla, Seville, Spain; 2 Servicio de Radiofísica, Hospital Infanta Luisa, Seville, Spain; 3 Servicio de Radiofísica, Hospital Virgen del Rocío, Seville, Spain; 4 Instituto de Biomedicina de Sevilla, IBIS, Sevilla, Spain; North Shore Long Island Jewish Health System, UNITED STATES

## Abstract

A model based on a specific phantom, called QuAArC, has been designed for the evaluation of planning and verification systems of complex radiotherapy treatments, such as volumetric modulated arc therapy (VMAT). This model uses the high accuracy provided by the Monte Carlo (MC) simulation of log files and allows the experimental feedback from the high spatial resolution of films hosted in QuAArC. This cylindrical phantom was specifically designed to host films rolled at different radial distances able to take into account the entrance fluence and the 3D dose distribution. Ionization chamber measurements are also included in the feedback process for absolute dose considerations. In this way, automated MC simulation of treatment log files is implemented to calculate the actual delivery geometries, while the monitor units are experimentally adjusted to reconstruct the dose-volume histogram (DVH) on the patient CT. Prostate and head and neck clinical cases, previously planned with Monaco and Pinnacle treatment planning systems and verified with two different commercial systems (Delta4 and COMPASS), were selected in order to test operational feasibility of the proposed model. The proper operation of the feedback procedure was proved through the achieved high agreement between reconstructed dose distributions and the film measurements (global gamma passing rates > 90% for the 2%/2 mm criteria). The necessary discretization level of the log file for dose calculation and the potential mismatching between calculated control points and detection grid in the verification process were discussed. Besides the effect of dose calculation accuracy of the analytic algorithm implemented in treatment planning systems for a dynamic technique, it was discussed the importance of the detection density level and its location in VMAT specific phantom to obtain a more reliable DVH in the patient CT. The proposed model also showed enough robustness and efficiency to be considered as a pre-treatment VMAT verification system.

## Introduction

The complexity of intensity modulated treatments in radiotherapy is increasing significantly, especially in rotational dynamic techniques, such as volumetric modulated arc therapy (VMAT), in pursuit of delivery demanding dose distributions in shorter treatment times with fewer monitor units (MUs) than conventional intensity modulated radiotherapy (IMRT) [1]. Nevertheless, the completely dynamic implementation involves an added complexity to the planning, since additionally to the dynamic multi-leaf collimator (MLC) movement, the dose rate and gantry motion have to be synchronized during irradiation [2]. New uncertainties about this dynamic nature of the VMAT delivery increase the complexity of the associated quality assurance (QA) [3–5]. Therefore, new QA systems are continuously becoming available, while also there exist no clear guidelines and criteria for the accuracy required.

It could be stated that, unlike static field IMRT, all systems implemented for VMAT QA have to face two main sources of uncertainty: one related to the dose calculation accuracy common to any modulated technique, and other linked to the continuous delivery of a discrete calculation.

On one hand, the dose calculation accuracy is a double problem concerning the consideration of patient heterogeneities and also the beam modifiers contribution to the final dose. On the theoretical definition of VMAT presented by Otto [2], Monte Carlo (MC) was already proposed as an effective and necessary tool [6, 7]. MC particle transport simulation is recognized for its higher accuracy to model linac heads, especially in non-standard dosimetric conditions, like the ones involved in VMAT treatments. In this way, it is possible to know the dose contribution of the scattered and transmitted radiation through the beam modifiers, which are expected to play a relevant role in a dynamic modulated technique, such as VMAT. Still considering the challenge of achieving operating times for clinical practice, the explicit and accurate calculation provided by MC method is suitable for assessing the real VMAT capabilities.

On the other hand, the accuracy of the dose distribution can also be compromised by potential differences between the discrete apertures and corresponding MUs proposed by the TPS, and those continuously delivered by the linac. Algorithms implemented in TPSs use discrete series of control points (CPs) to optimize VMAT treatments in such a way that the quality of the final solution depends on the number of these starting points [8]. In spite of the dynamic behavior is correctly modelled in some TPSs, even using Monte Carlo dose calculation as Monaco^®^, most planning systems make an approximation by summing doses calculated at the discrete CP and not in between [9]. This means that the MUs optimized for a fixed aperture shape are actually delivered with different shapes at different angles [10]. For this reason, the linac log files registered during the irradiation are usually considered to compute the delivered dose distribution. The considerable data recorded in log file requires a reduction of the actual dynamic event for the subsequent calculation. This reduction imposes a level of discretization that can be equivalent to the considered level in the planning system [11, 12], but it seems reasonable to think the larger the number of CPs calculated from the log file, the better this approximation is.

In order to cover both type of uncertainties commented above, some works proposed MC simulation of linac log files recorded during treatment delivery [6, 13, 14]. The dose distribution discrepancy introduced between the discretized plan and the continuous delivery was assessed by incorporating DynaLog files into MC simulations for RapidArc QA [6]. For that work, a new DOSXYZnrc source [15] was used to compute the dose distribution, by considering a continuous variable beam configuration, through sampling-based methods. This approach reached simulation times for routine clinical applications, although the required statistical uncertainty was only ensured in the high dose voxels. It could be efficient for treatment verification but not suitable to assess one of the supposed benefits of VMAT associated to the reduction of integral radiation dose to the rest of the body [1]. Furthermore, this approach may over-simplify VMAT delivery in certain parts of the arc where changes in gantry speed are larger than in others and the variable dose rate could not be considered with the same accuracy. This could be important to assess the potential radiobiological influence of different dose rates during VMAT delivery. Because of these considerations, other works incorporate different methods to represent the linac motion with a VMAT delivery emulator [14], where important differences were found between static and continuous dose calculation, as it was differently reported by Teke et al. [6]. It is important to remark that different results could be also linked to the type of verification systems implemented to assess the impact of VMAT delivery efficiency, since dose experimental measurements should be used to support the dose calculation from log files, or even from an emulator. Actually, these approaches showed only an accurate second check of dose calculation based on MC by considering the delivered geometrical parameters, since no experimental measurements were directly included to estimate the actual dose dynamically delivered to the patient. For this purpose, the discretization degree and the accuracy used in dose calculation would be sensible to the efficiency of detectors and their locations inside VMAT systems.

Several studies have stated poor correlation between the most commonly employed gamma index for comparing dose distributions in a phantom and dose errors in the patient anatomy [16, 17]. In addition, this gamma index can be misleading and insensitive to clinically relevant dosimetric errors. Therefore, dose-volume histogram (DVH)-based metrics should also be examined, especially in regions with high-dose gradients [18]. Apart from singular solutions for own use [19], only a limited number of measurement-based 3D anatomy dose QA devices are commercially available: 3DVH (Sun Nuclear Corporation), COMPASS (IBA Dosimetry), Delta4DVH Anatomy (ScandiDos). These are associated with diode arrays with different geometries, capable of provide 3D dose verifications [20, 21], or planar measurements obtained by an ion chamber array mounted on the gantry [22]. Detector arrays limited by their spatial resolution, may affect the verification results due to under-sampling effects [23]. The Delta4DVH Anatomy and COMPASS use independent dose calculation algorithms, which calculate the dose to the patient using the energy fluence. The 3DVH does not recalculate the dose, but only perturbs the TPS patient planned dose to account for known errors measured in the conventional QA [24]. In this case, the limitations related to the dose calculation engine are still present.

An accurate 3D verification of VMAT with high calculation resolution, based on the information provided by the log file, demands the implementation of an experimental validation with high detection degree in order to minimize potential spatial mismatching between calculation and measurements. Although the data analysis process makes film dosimetry a less popular method for QA compared to previously mentioned verification systems, the high spatial resolution, minor energy dependence, and near tissue-equivalence provided by the radiochromic films, are well suited for VMAT QA purposes. The use of film dosimetry in a 3D spatial distribution was already proposed [25, 26], but the measurements were not managed to reconstruct the 3D dose distribution in the patient anatomy.

In order to evaluate and address the limitations inherent to VMAT verification systems discussed above, we present a model based on the MC simulation of log files with experimental feedback from film dosimetry. This proposal is focused to provide a tool for general VMAT evaluation, more than to present another alternative for clinical routine, although the model could be also used for it. The sampling of CPs is performed according to dose rate changes and is able to consider different discretization levels. The corresponding MUs are experimentally obtained from radiochromic films rolled at different radial distances in a cylindrical phantom, called QuAArC, specifically designed to reconstruct a DVH by considering an approximation of entrance fluence, 3D relative dose distribution and absolute dose. The high detection density provided by the film ensures the agreement between the considered CP and detector location.

## Materials and Methods

### QuAArC system

**Fig 1:**
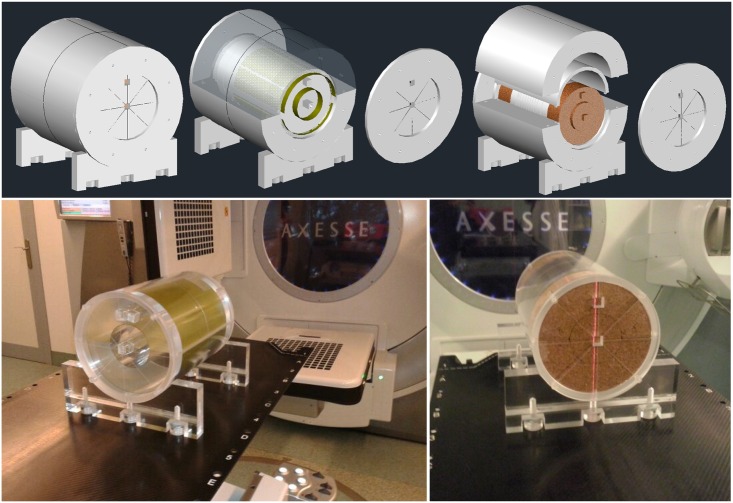
QuAArC phantom. Different setups of QuAArC phantom prototype (top) and the final PMMA phantom (bottom). On the top, the big setup is depicted with radiochromic films rolled at two different radial distances. The independent inside part of the phantom is shown on the bottom, and constitutes the small setup.

A cylindrical shape PMMA phantom (physical density 1.19 g/cm^3^), consisting of a set of two concentric cylinders, was specifically designed to host radiochromic films rolled at different radial distances from the isocenter, for a 3D and continuous dosimetric verification. As it can be seen in Fig 1, different radial distances were selected to obtain dose distributions close to the treatment site and also estimation of fluence at the beam entrances in the phantom. In order to ensure the films are correctly placed, the cylinders size was thought to be equal to the length of films. Moreover, the phantom has several marked reference lines to know the exact film location during the setup mounting and positioning on the treatment table with the usual laser system (Fig 1). Other components allow a configuration prepared for axial or coronal films and dose point measurements with several types of ion chambers at different locations (Fig 1). Besides the PMMA components, it also includes a set of cork cylinders and inserts to simulate lung or air-like cavities. In order to consider the verification of several treatment regions, QuAArC phantom comprises two different setups: one with dimensions of 30 cm diameter and 30 cm length (big setup), and the other with 20 cm diameter and 28 cm length (small setup).

For QuAArC verification of real plans, Gafchromic EBT3 films with dimensions of 20.3 x 25.4 cm2 were rolled around the outer cylinder, at 1 cm depth (2 films), and the inner cylinder, at 6 cm depth (1 film) in the phantom, from now on the outer and inner film scrolls, respectively. Absolute dose measurements with a CC04 ion chamber (IBA Dosimetry) placed at the isocenter for each treatment in QuAArC phantom were also performed. Chamber reading conversion to dose was made following the IAEA TRS-398 protocol, and then compared to the corresponding absorbed dose to water calculated by MC in the PMMA QuAArC phantom. The irradiated films were processed following a specific protocol, which included the characterization procedure of the scanner-film system, in order to minimize the related uncertainties. All films were scanned at least 12h after exposure, using an Epson Expression 10000 XL (Seiko Epson Corp.) flatbed scanner at a resolution of 75 dpi and a depth of 48-bit RGB, without applying any color correction. The films were all scanned in the portrait orientation at the center of the scanner to use its optimum part, which was determined through the characterization process. A calibration curve for a single batch of EBT3 and 6MV photon beams was obtained by irradiating sixteen pieces of 5 × 6 cm^2^ from the same film. The pieces were individually irradiated with a 10 x 10 cm^2^ beam in reference conditions, with doses ranging from 0 to 400 cGy, for an appropriate characterization of the film response behavior, including more than 12 points as proposed by Bouchard et al. [27]. Because of film scrolls normally would receive lower doses, especially in outer films, than the films used in typical verifications, it was important to have an exhaustive characterization of dose-response curve in the low-dose range. For the conversion of the film pixel value into dose, a multichannel method was used [28], and also corrections for the non-uniformity lateral dose dependence response of the scanner were applied to the three channels (RGB). Although the same effect in the longitudinal scanner direction was also characterized, corrections were not applied since it was found to be negligible.

### MC simulation and discretization process of log files

For a QA model applied to VMAT evaluation, an automated MC simulation of the geometry of every control point was implemented in an in-house developed Matlab program. Simulation parameters were extracted from the log files recorded during the treatment delivery by means of specific software written in C++ developed by our group. This software allows the communication with the Elekta linac in real time, under the iCom Protocol, and is able to record all the CP parameters every 0.25s or 1s during beam on or beam off, respectively. This 4 Hz recording rate is similar to other tools implemented for log file analysis in Elekta linacs [11]. The analysis and comparison of relevant delivery parameters can always be made without further reduction of the data recorded in these log files. However, the data retrieved for MC simulation and dose calculation from log files are dependent on the required discretization level. The implementation used for QuAArC system applies a discretization method designed for taking into account the relationship between changes in MUs and gantry motion for a higher sampling rate when more significant changes are present in a specific sector of the arc. In this way, the dose rate is intrinsically considered for the sampling process. Obviously, this process does not lead to equi-spaced CPs along the arc. For this work the delivered parameters for MC simulation were considered with more CPs describing the arc (fine log) than the one usually presented in the DICOM-RT plan file from the TPS (coarse log). For the coarse discretization level, after excluding CPs where there was no variation in cumulative MU, the actual cumulative value at the end of each control point was retrieved along with the actual leaf positions and the corresponding gantry angle, recorded during that sampling time. In general, the number of simulated CPs will be dependent on the original treatment plan and its complexity. In particular, this number was about three times higher for the fine approach compared to the coarse one for the evaluated plans in this work.

The EGSnrc Monte Carlo user code BEAMnrc [29] was used to simulate the 6 MV photon beams from two Elekta linacs, Axesse and Synergy. The following BEAMnrc/EGSnrc transport parameters were employed: NIST for bremsstrahlung cross sections; EXACT as boundary crossing algorithm and PRESTA-II as electron-step algorithm; for bremsstrahlung angular sampling, the leading term of Koch-Motz distributions was chosen; electron and photon cutoff energies were 0.7 MeV (0.189 MeV kinetic energy) and 0.01 MeV, respectively. For all simulations, electron range rejection with an energy cutoff of 2.0 MeV was implemented. For the MC simulations of VMAT treatments, a phase-space data (PSD) file was first obtained from the simulation of the corresponding linac head non-treatment dependent, in order to be used as source for the transport simulation through the geometry of beam modifiers specific to each case. The subsequent PSD files were obtained by means of the simulation of each CP geometry considered from the log file and were scored at the exit of the corresponding linac head to be used as inputs for the dose calculation in the patient.

To accurately simulate the beam modifiers contribution, MLC geometry was modelled using the BEAMnrc MLCE component module. This linac model has been previously validated for other published works [30] by comparison with experimental measurements of profiles and depth dose distributions corresponding to several field sizes (ranging from 2.4x2.4cm^2^ to 16x21cm^2^) obtained with ionization chamber and semiconductor diode. These results were achieved with a statistical uncertainty of less than 1%, and an agreement within 2% was obtained between experimental measurements and MC calculations. Furthermore, experimental measurements with radiochromic film were included to validate inter and intra leaf transmission with a nominal gap and a slight leaf bank tilt of the focused leaves.

All MC simulations were distributed on a cluster of four 12-core 2.19 GHz CPUs AMD Opteron.

### Dose processing in QuAArC and MU adjustment for 3D dose reconstruction

The corresponding dose calculation was carried out from the phase-space files previously obtained for each CP, by means of BEAMDOSE, an in-house DOSXYZnrc code modification. This code allows knowing every aperture contribution to each voxel in order to score the individual dose through each voxel of the phantom representing either a patient CT or a QA phantom. Dose calculation was performed with a high resolution grid, consisting on 256 × 256 voxels per slice, for a fair comparison with film. For these simulations, particle transport parameters were similar to those used for the BEAMnrc simulations, except the energy threshold for electron transport ECUT, which was set to 0.512 MeV, considering the voxel size. The number of histories used was selected to ensure the statistical uncertainty below 1% in the final dose for all the voxels inside the treatment region. The same statistics was achieved independently of the discretization level implemented by considering different number of history cases according to the number of CPs from the log file. In order to process and evaluate the unusual dose distribution in the irradiated film scrolls, specific in-house software was also developed in Matlab, which incorporates the analysis of dose distributions, profiles, dose difference maps, and 2D/3D gamma index. The cylindrical distribution of the films in the 3D dose cubic voxelized matrix (1.25 x 1.25 x 1 mm^3^) demands a specific recruitment process based on interpolations each 0.5° between voxel values taken from the nearest neighbors in the three axes. In order to take into account the disagreement between different coordinate systems, one planar MC matrix was reconstructed for each, inner and outer scrolls, from 5 planar matrices generated by shifting the isocenter to ± 1 pixel. In this way, we can assume that the uncertainty location between MC scroll and film scroll was ± 1.25 mm, for the considered grid. During the comparative analysis between both dose distributions, an efficient non-deformable mutual information method was implemented in our software to account for small shifts or rotations that could take place during the film processing.

In order to obtain an experimental DVH, a least-squares optimization method following the expression (1) was implemented in our software, taking into account the measurements obtained with the rolled films and the absolute point dose in the process.

$$\underset{x}{min}\frac{1}{2}\|C\cdot x-d{\|}_{2}^{2}\;such\;that\{\begin{array}{c} A\cdot x\leq b\\ Aeq\cdot x=beq\\ lb\leq x\leq ub \end{array}$$ (1)

*C* is the MC dose matrix containing the individual CP contribution to each voxel; *x* is a MU weight vector, considered as a percentage of MU change from either the initial solution calculated from the log file or the final proposed solution; *d* is the matrix composed by the films dose matrices and the isocenter absolute dose measured with ion chamber; *A* and *b* are the linear inequality constraint to establish the tolerance of dose difference between MC dose and measurements (considering both, positive and negative differences); *Aeq* and *beq* are the linear equality constraints, specified as a vector and a scalar, respectively, which represent the original MU weight vector from log file, and the total treatment MU; *lb* and *ub* represent the lower and upper bounds for the solution *x*, allowing the control of the change level on the MU values from log file to match the experimental value. In this process, the global contribution of each individual CP to the whole treatment is assessed and adjusted according to the measurements. The rolled films provide us measurements of fluence estimation (outer film scroll) and relative dose contribution (inner film scroll) of the direct entrance, lateral overlapping, and the opposite irradiation for the whole arc. The contribution of opposite irradiation present in these film scrolls, it is not a handicap because it is considered in a global manner along the optimization process. It is important to note that the values *lb* and *ub* are considered as a percentage of the original MU corresponding to each CP in the log file. In this way, it is possible to accept only relative small changes for each new MU during the iterations in the optimization process. The latter in addition to a minimum tolerance of dose difference, makes possible to obtain experimental values as a result of an average of the heterogeneity within the irradiated area in the film corresponding just to one CP. Therefore, the adjustment is mainly performed by using the contribution from lateral overlapping, what is directly related to the discretization effect.

This approach makes possible the study of the effect of considering several discretization levels from the log file simulation, since deviations measurement caused by a mismatching detection location can be overcome thanks to the high density detection inherent to film dosimetry.

On the other hand, the control of parameters in this feedback process would also allow obtaining a new proposal of a treatment plan with larger changes in MU values, but still in accordance to the experimental measurements able to provide a final DVH on patient CT clinically acceptable. This last operative option of our method was not evaluated for this work.

**Fig 2:**
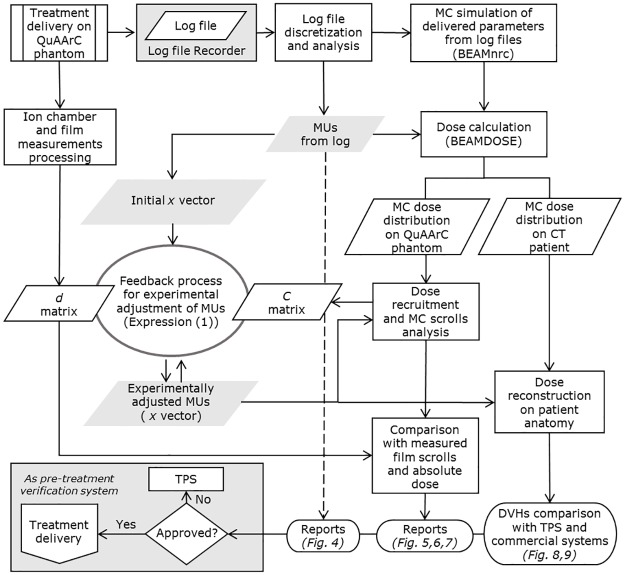
Flowchart describing the proposed model. Film measurements in QuAArC phantom are implemented in a feedback process in order to adjust the MUs from the log file for reweighting the full MC simulated CPs to obtain the final reconstructed DVH on patient CT.

As a summary, a general flowchart representing the proposed model is shown in Fig 2.

### QuAArC system validation with clinical cases

As a proof of concept of our model, four clinical cases, corresponding to prostate and head and neck (H&N) treatments were selected. For the real clinical application, these cases were planned with two different commercial TPSs, Monaco, Elekta and Pinnacle, Philips, and verified with ScandiDos Delta4 and IBA COMPASS systems, respectively.

For the Monaco/Delta4 option, two cases with two different plans each one were evaluated. A first solution for both cases (plan A), did not meet the acceptance criteria, which consisted of more than 95% of the evaluated points with a global gamma index < 1, for 2.5% dose difference (DD) and 2mm distance to agreement (DTA) criterion and a dose threshold of 20%, in the Delta4 detector planes. A second solution (plan B) was proposed for both cases, in order to find solutions that meet these acceptance criteria when verified with Delta4. In particular, the plan A for both cases, prostate and H&N, failed with a passing rate of 91.8% and 76.9%, respectively, while the plan B passed with 95.6% and 99.6%, respectively.

From a pool of clinical cases, the prostate case was selected for this study due to the high similarity between the DVHs presented by Monaco TPS solutions for both plans, A and B, which consisted of a single arc VMAT treatment composed by 87 and 78 CPs, respectively. The H&N case consisted of a boost phase treatment. In this case, a single arc VMAT plan with 93 CPs was the plan A, and the second plan proposed (plan B) was a static IMRT technique, which did pass the Delta4 QA and was accepted for treatment. This IMRT plan, consisting of 34 segments distributed in 9 incidence angles (ranging from 205° to 180° CW), and it was specifically considered as a static example to check the correct implementation of our algorithm, since potential discrepancies between MC and the film scrolls generated by the discretization process would not be present in this scenario.

For the Pinnacle/COMPASS option, also a prostate and H&N cases were selected, although only two VMAT treatment plans were included, both having met the acceptance criteria. These criteria were based on the DVHs comparison between Pinnacle TPS and COMPASS solutions, through relevant dose metrics. These VMAT plans consisted of a single arc with 90 equi-spaced CPs treated with a hypofractionation scheme (3Gy/fx), for the prostate case, while for the H&N case consisted of a double arc with a total of 180 equi-spaced CPs.

## Results and Discussions

### Effect of discretization level on treatment verification evaluated with QuAArC

**Fig 3:**
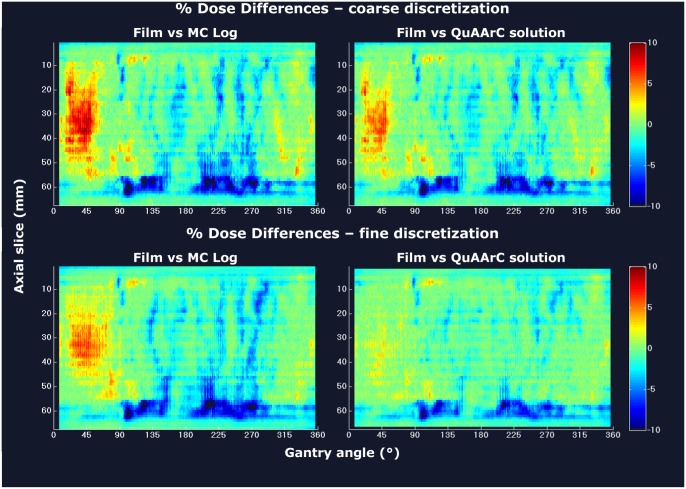
Effects of considering a different discretization level from the log files. Percent dose difference matrices of the inner film scroll versus MC Log (left) and versus the corresponding QuAArC solution (right) for coarse discretization (top) and fine discretization (bottom), corresponding to prostate VMAT plan B.

**Fig 4:**
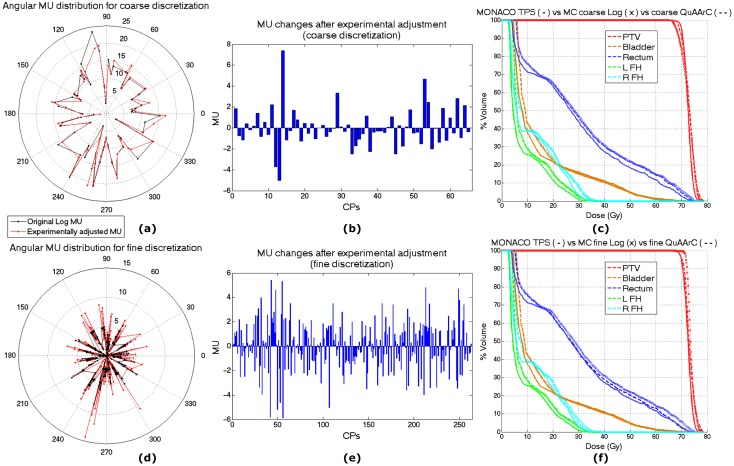
QuAArC system reports for fine and coarse discretization. Comparison between original and experimentally adjusted solutions for both discretizations. Angular MU distributions from the original log file MC simulation and the experimentally adjusted with QuAArC (a) and the corresponding MU differences for coarse discretization (b). The same for fine discretization (d and e). DVHs comparison between Monaco TPS solution, MC log file simulation and QuAArC reconstructed solution, for coarse (c) and fine discretization (f). All for prostate VMAT plan B.

Potential effects of considering a different discretization level from the log files for MC simulation in treatment verification was analyzed by comparison with the film scrolls irradiated in the QuAArC phantom (Fig 3). These effects can be observed in the corresponding DVHs experimentally reconstructed on the patient CT data (Fig 4(c)–4(f)) by means of the model described in previous section. Although in the Figs 3 and 4, only results for prostate VMAT plan B are shown, the following considerations can be extended to the other plans. As expected, the more pronounced discrepancies between MC log calculation and film were found for the coarse discretization, as it is shown in the left column of Fig 3. Furthermore, the different level of discretization between coarse and fine also had an impact on the procedure for obtaining the dose distribution experimentally reconstructed (QuAArC solution) from the measurements in the QuAArC phantom, as it can be observed in the right column of Fig 3. This shows how our model could establish the required level of discretization to obtain an adequate VMAT verification based on MC simulation of log files free-dependent on detection density. It is important to remark that these differences would not have been so evident whether a lower density detection implemented in other VMAT verification systems would have been employed. On the other side, the consistency of these results provides confidence on our model.

After experimental adjustment, the more relevant MU changes were found at the same arc locations for both discretization levels, showing the procedure does not work randomly along the whole arc, but the algorithm proposed bigger changes where the differences between theoretical and experimental values were higher. Also, as expected, the global change was more uniformly distributed along the arc for the fine (Fig 4(d) and 4(e)) than for the coarse discretization (Fig 4(a) and 4(b)). This latter showed that the MU adjustment was mainly carried out with the lateral contribution from the contiguous CPs, what was our goal in order to achieve a reconstruction of the accumulated MU in the log file with the information continuously registered in the film.

According the comparison followed in Fig 3 with the rest of measurements in the phantom, the finer approach provided a more reliable reconstructed DVH solution (Fig 4(c)–4(f)), and was considered as the necessary option in QuAArC verification procedure for this case. Although for this case, coarse QuAArC approach provided a similar DVH solution that could have also been approved, the same was not observed for the rest of analyzed cases. Anyway, the evaluation of more clinical cases would be necessary to prove what level of discretization could be enough for an efficient verification procedure in shorter times. With this work, we suggest that this kind of studies with verification systems different to the model proposed here, could be biased due to the use of lower detection density and to different spatial distribution.

### Proof of concept of the experimental feedback process

**Fig 5:**
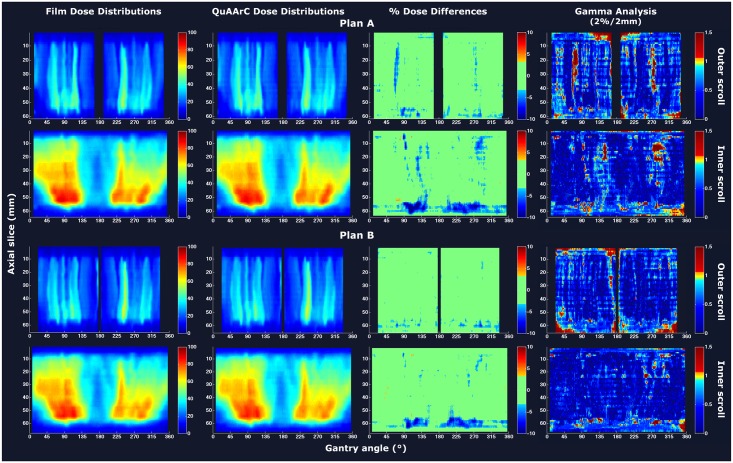
Proof of concept of the experimental feedback process for the prostate case from Monaco. Film and QuAArC dose distributions, corresponding percent dose differences and gamma analysis are shown for outer and inner scrolls for VMAT Plan A (first and second rows, respectively), and the same for VMAT Plan B (third and fourth rows).

**Fig 6:**
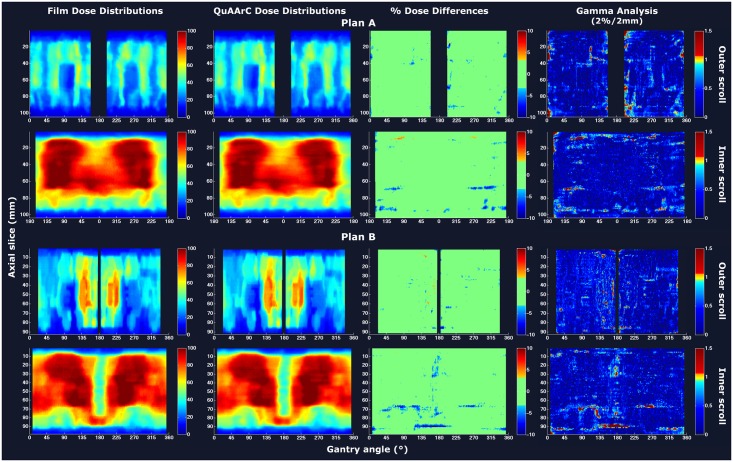
Proof of concept of the experimental feedback process for the H&N case from Monaco. Film and QuAArC dose distributions, corresponding percent dose differences and gamma analysis are shown for outer and inner scrolls of VMAT Plan A (first and second rows, respectively), and the same for IMRT Plan B (third and fourth rows).

**Fig 7:**
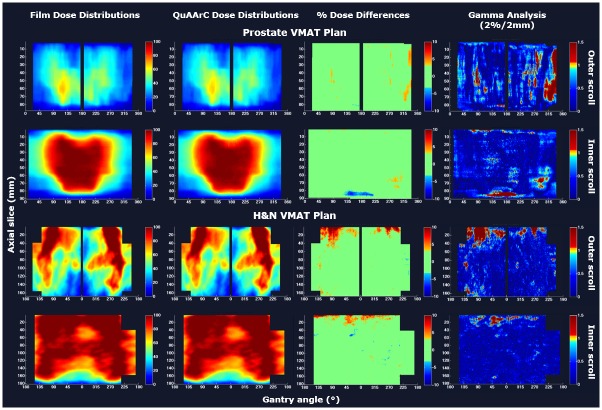
Proof of concept of the experimental feedback process for the prostate and H&N VMAT plans from Pinnacle. Film and QuAArC dose distributions, corresponding percent dose differences and gamma analysis are shown for outer and inner scrolls.

As a part of the proof of concept of the model, comparisons of outer and inner film scroll dose distributions with the ones obtained by means of QuAArC system were carried out (Figs 5–7), for prostate case and H&N case, respectively. The color code used to represent the percent dose difference matrices was set according to the passing rate values presented in Table 1 (% pixels having a dose within 3%). Gamma analysis based on 2% DD/2mm DTA criteria was also included. All QuAArC scrolls showed a high agreement with the measured film scrolls for both, percent dose differences and gamma analysis, which values are given in table 1. Since there is always a limitation regarding the discrete calculation, even with the fine discretization under consideration, minor differences were assumed. Anyway, these small differences observed were mostly located outside the treatment field or at the edges. Note that this comparison was carried out with the MC scrolls resolution (1 x 0.7854 mm^2^ for outer scroll and 1 x 0.3523 mm^2^ for inner scroll) obtained after the dose recruitment described in Materials and methods section.

**Table 1:** Summary of absolute doses, percent dose differences and gamma index passing rates for all evaluated treatment plans.

Plan	Absolute dose (Gy) (% deviation)			Dose difference passing rates (%) (<3%)				γ-index passing rates (%) (2%/2mm)			
				Outer scroll		Inner scroll		Outer scroll		Inner scroll	
	CC04	MC LOG	QuAArC	MC LOG	QuAArC	MC LOG	QuAArC	MC LOG	QuAArC	MC LOG	QuAArC
**Prostate Case from Monaco**											
**A**	2.48	2.51 (1.21)	2.53 (2.02)	92.95	91.94	78.33	84.85	90.77	90.12	87.91	93.19
**B**	2.45	2.42 (-1.22)	2.45 (0.00)	92.78	95.48	80.83	91.19	88.34	92.66	89.94	97.09
**H&N Case from Monaco**											
**A**	2.02	2.01 (-0.49)	2.06 (1.98)	96.89	97.61	89.52	96.04	96.28	97.45	91.19	98.18
**B**	2.06	2.07 (0.48)	2.07 (0.48)	97.53	98.16	93.16	94.51	97.87	98.40	96.84	97.60
**Prostate case from Pinnacle**											
**-**	3.67	3.56 (-3.00)	3.57 (-2.72)	99.05	99.53	97.55	96.63	96.51	90.53	95.45	96.62
**H&N case from Pinnacle**											
**-**	2.03	2.13 (4.93)	2.16 (6.40)	77.08	90.78	80.43	94.16	78.64	91.90	82.78	97.84

Table 1 shows a summary of passing rates for dose differences within 3% and global gamma index with 2%/2 mm criteria obtained for MC simulations of fine log discretization (MC LOG) and the corresponding QuAArC solutions after experimentally adjustment. The values for the absolute dosimetry performed with ion chamber in QuAArC phantom and the obtained from MC LOG and QuAArC solutions, after experimental adjustment, were also included in Table 1. For Monaco plans, all MC LOG and QuAArC absolute dose values were obtained with less than 1.25% of statistical uncertainty, and agreed with the experimental measurement within 2%. For Pinnacle plans, the prostate VMAT case agreed well within the 3% in absolute dose, while the H&N VMAT plan presented a higher deviation, although, it was not significant for reconstructing the DVH, so we considered this result acceptable. For this case, a location less exposed to high dose gradient should have been chosen for the absolute dose point measurement in order to have a more reliable experimental value. For further evaluation one should consider repeat this measurement, but the high agreement with film and improved results for QuAArC compared to the MC LOG, were considered to be sufficiently acceptable to obtain a reconstructed DVH, which better estimates the delivered dose. In general, the passing rates improved for QuAArC solution after experimental adjustment regarding the MC LOG in both, 3% dose difference and gamma analysis. For QuAArC solution, all evaluated plans had a γ index < 1 passing rate greater than 90% using 2%/2 mm criteria in both scroll regions (outer and inner) and greater than 95% most of them. For 3%/3 mm criteria, passing rates were greater than 97%, in all cases. As expected, this same test based on coarse discretization approach provided worse passing rates.

For the IMRT H&N plan B (4^th^ row of Table 1), the results were practically the same for MC LOG and QuAArC solution, meaning that our model did not modify the MUs when the delivery was static.

### QuAArC application to clinical cases

**Fig 8:**
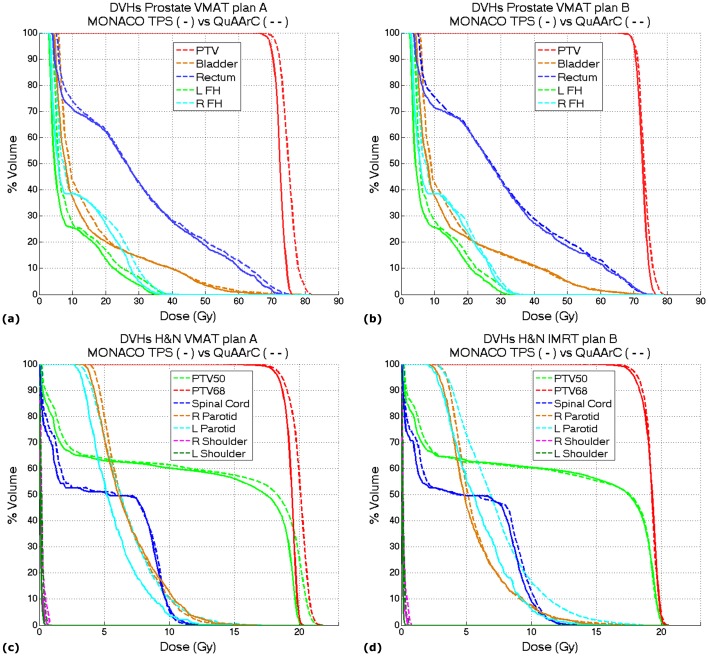
DVHs comparison between Monaco TPS solution and QuAArC reconstructed solution. VMAT plan A (a) and B (b), for the Prostate case and VMAT plan A (c) and IMRT plan B (d), for the H&N case.

**Fig 9:**
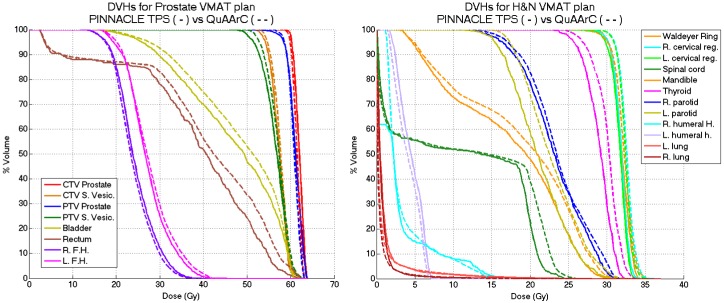
DVHs comparison between Pinnacle TPS solution and QuAArC reconstructed solution. Prostate VMAT plan (left) and H&N VMAT plan (right).

In the case of considering our QuAArC model as a pre-treatment VMAT verification system, we propose the reconstructed DVH by means of our model as the relevant metric for acceptance criteria of the treatment planning under evaluation (Figs 8 and 9).

Starting the discussion with the cases from Monaco (Fig 8), it is important to remark that the commented agreement for the static IMRT case was also observed in the DVHs comparison with Monaco TPS solution (Fig 8(d)), since Monaco calculation is strongly based on MC, what is similar to our full MC model. The minor discrepancies in OARs could be due to the different consideration of beam modifiers contribution to the dose.

In the static plan, the MC log simulation represented the measurement well enough, but it was not the same for VMAT plans, as it can be seen in Table 1. MU adjustment approach proposed in our system showed to be necessary for exhaustive dynamic treatment verification. Apart from the IMRT commented above, the VMAT cases which were not accepted with Delta4 verification, were well adjusted by our model (plan A in Figs 5 and 6), although the resulting DVHs (Fig 8(a)–8(c)) showed to be significantly different to the planning with Monaco TPS, while the DVH corresponding to the VMAT plan B accepted with Delta4 (Fig 8(b)) showed to be very similar to the Monaco solution. These results obtained with our model showed to be in tune with Delta4 verification. In any case, considering the two uncertainties pointed out in the Introduction section, it could be estimated that the potential discrepancies involved in the continuous delivery of a discrete calculation are more significant than those due to the dose calculation accuracy.

For the cases planned with Pinnacle TPS by implementing a non MC-based algorithm dose calculation, the same DVHs comparison is presented on Fig 9. These plans showed more differences for the OARs, as opposite to what was observed for Monaco plans. This discrepancy is assumed to be due to the different algorithms used for dose calculation. Pinnacle can be underestimating the transmission contribution from the MLC to the final dose. Anyway, in general, the DVHs obtained with QuAArC for the target planning volumes showed similar results to the plans calculated with Pinnacle, likely because these plans were approved by COMPASS. Otherwise, more differences could be found.

It is necessary to remark that all these results in Figs 5–7 and Table 1, indicated that the model developed was robust and consistent, and were included in this work only to prove the feasibility of the novel feedback procedure and to provide confidence about the experimental reconstruction of DVHs (Figs 8 and 9). In fact, these DVHs should be considered as the main evaluation metric for a potential use of QuAArC as a VMAT verification system.

## Conclusions

The developed QA model allowed the verification of VMAT treatments with high accuracy provided by the MC explicit radiation transport simulation of the actual delivery treatment parameters from the log files, and with the high spatial resolution provided by film dosimetry. The proposed model is able to control and reduce the uncertainties involved in complex dynamic techniques, what is useful for further studies about VMAT efficiency versus static IMRT techniques for specific clinical cases, and also for carrying out the linac commissioning and evaluation of other QA systems.

This work was not focused to make a comprehensive study on the different verification systems and dose calculation algorithms for dynamic techniques. Anyway, few clinical cases were evaluated to check the feasibility of our model. On one hand, for those cases which were approved with Delta4 and COMPASS, QuAArC system provided similar DVHs to the solutions from TPSs corresponding to planning targets, Monaco and Pinnacle, respectively. More important disagreement was observed for DVHs corresponding to OARs in the cases from Pinnacle/COMPASS. The dose calculation uncertainty using Monaco TPS was observed to be not as relevant as the uncertainty linked to the dynamic delivery. However, greater differences were found when QuAArC solutions were compared with Pinnacle TPS, where this uncertainty, linked to the dose calculation accuracy, also added discrepancies, as expected. On the other hand, for those cases which were not previously approved with Delta4 from Monaco TPS solutions, QuAArC did show a greater disagreement for DVHs of PTVs. All these results proved that QuAArC system was consistent with expected results, what support the viability of the model for this kind of studies.

It is important to note that the QuAArC phantom based on film can be implemented apart from full MC log calculation, whether the TPS is able to provide individualized CP dose contribution. This would lead to more efficient computational times for routine pre-treatment verification, although with our approach based on MC calculation, the results were more reliable and, in fact, they were ready at time of film processing stage.

Besides the effect of dose calculation accuracy of a dynamic technique, this work was mainly focused on the evaluation of the effect of the detection density level and its location in a specific phantom to obtain a more reliable DVH. This could be useful to detect potential wrong decisions based on the results from commercial VMAT verification systems, due to mismatching between control points used for dose reconstruction and the detector locations. This latter aspect is being evaluated by our group in a current project in which, we would like to include as many verification systems as possible. QuAArC system is also being adapted for 4D verification to adequately verify treatments such as stereotactic body radiotherapy (SBRT) for lung cancer.

## Supporting Information

S1 Fig QuAArC phantom. (TIF)

S2 Fig Flowchart describing the proposed model. (TIF)

S3 Fig Effects of considering a different discretization level from the log files. (TIF)

S4 Fig QuAArC system reports for fine and coarse discretization. (TIF)

S5 Fig Proof of concept of the experimental feedback process for the prostate case from Monaco. (TIF)

S6 Fig Proof of concept of the experimental feedback process for the H&N case from Monaco. (TIF)

S7 Fig Proof of concept of the experimental feedback process for the prostate and H&N VMAT plans from Pinnacle. (TIF)

S8 Fig DVHs comparison between Monaco TPS solution and QuAArC reconstructed solution. (TIF)

S9 Fig DVHs comparison between Pinnacle TPS solution and QuAArC reconstructed solution. (TIF)

S1 Table Summary of absolute doses, percent dose differences and gamma index passing rates for all evaluated treatment plans. (PPTX)
