# Correction: 3D VMAT Verification Based on Monte Carlo Log File Simulation with Experimental Feedback from Film Dosimetry

**DOI:** 10.1371/journal.pone.0172378

**Published:** 2017-02-13

**Authors:** A. R. Barbeiro, A. Ureba, J. A. Baeza, R. Linares, M. Perucha, E. Jiménez-Ortega, S. Velázquez, J. C. Mateos, A. Leal

The following information is missing from the Funding section: This work was supported by the Andalusian Health Service (reference project CTS 2482).
