# Supplementary figures and images for: 3D VMAT Verification Based on Monte Carlo Log File Simulation with Experimental Feedback from Film Dosimetry
Source: PLoS One. 2016 Nov 21;11(11):e0166767. doi: 10.1371/journal.pone.0166767 (PMC5117721; doi:10.1371/journal.pone.0166767)

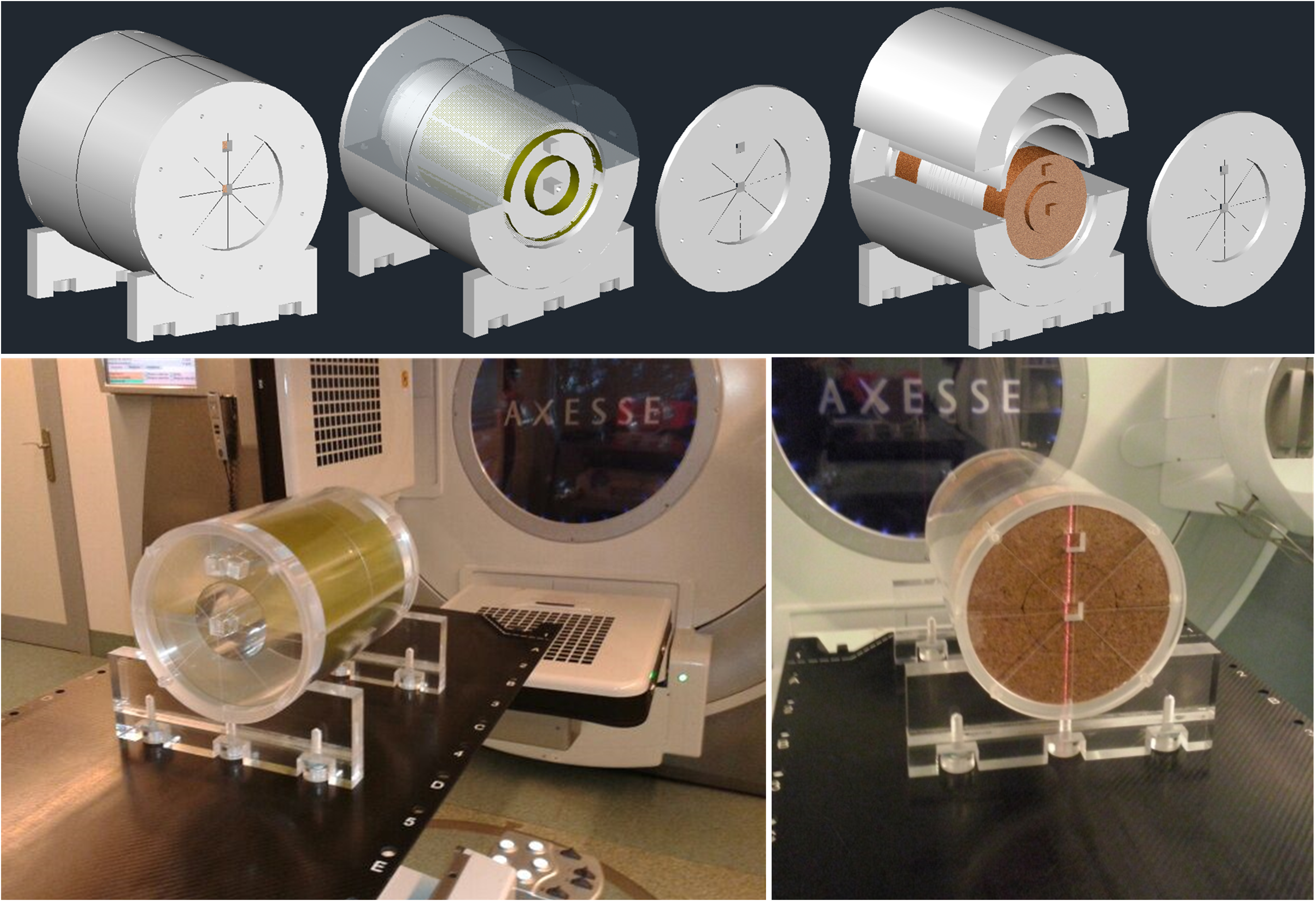

Supplement: S1 Fig — (TIF) [file pone.0166767.s001.tif]

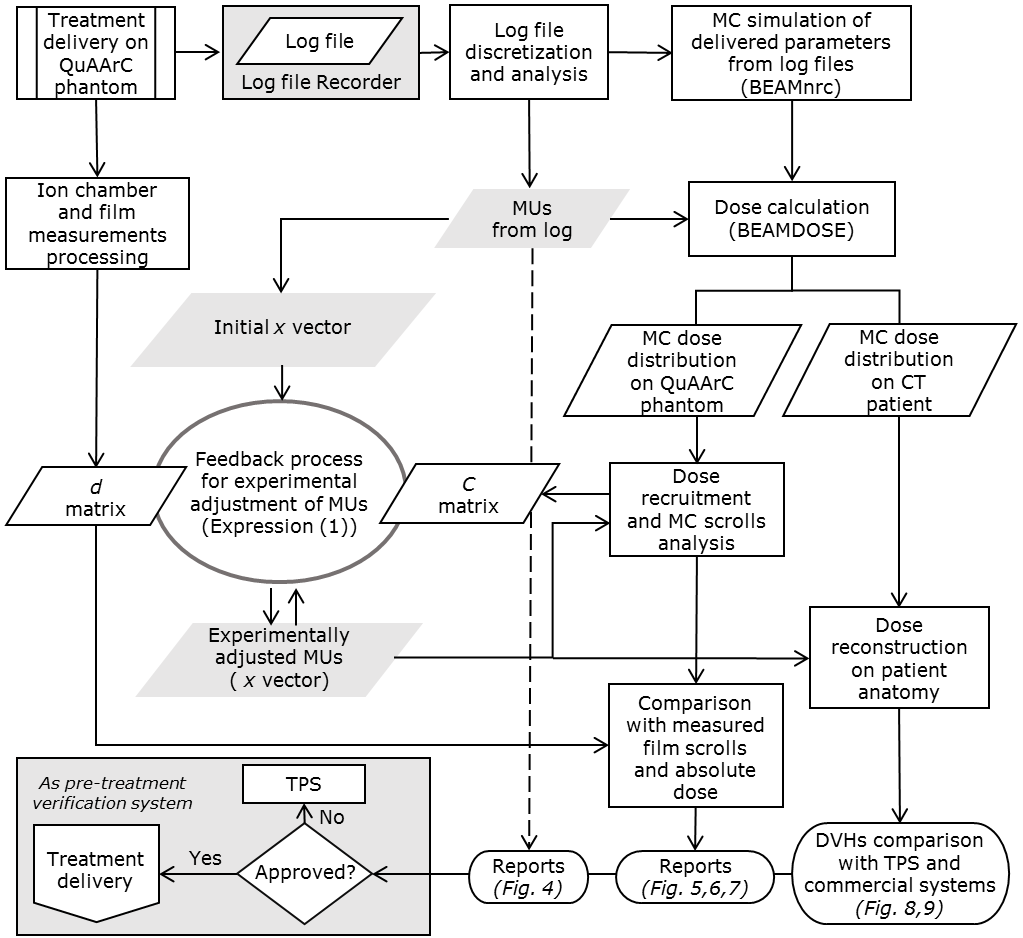

Supplement: S2 Fig — (TIF) [file pone.0166767.s002.tif]

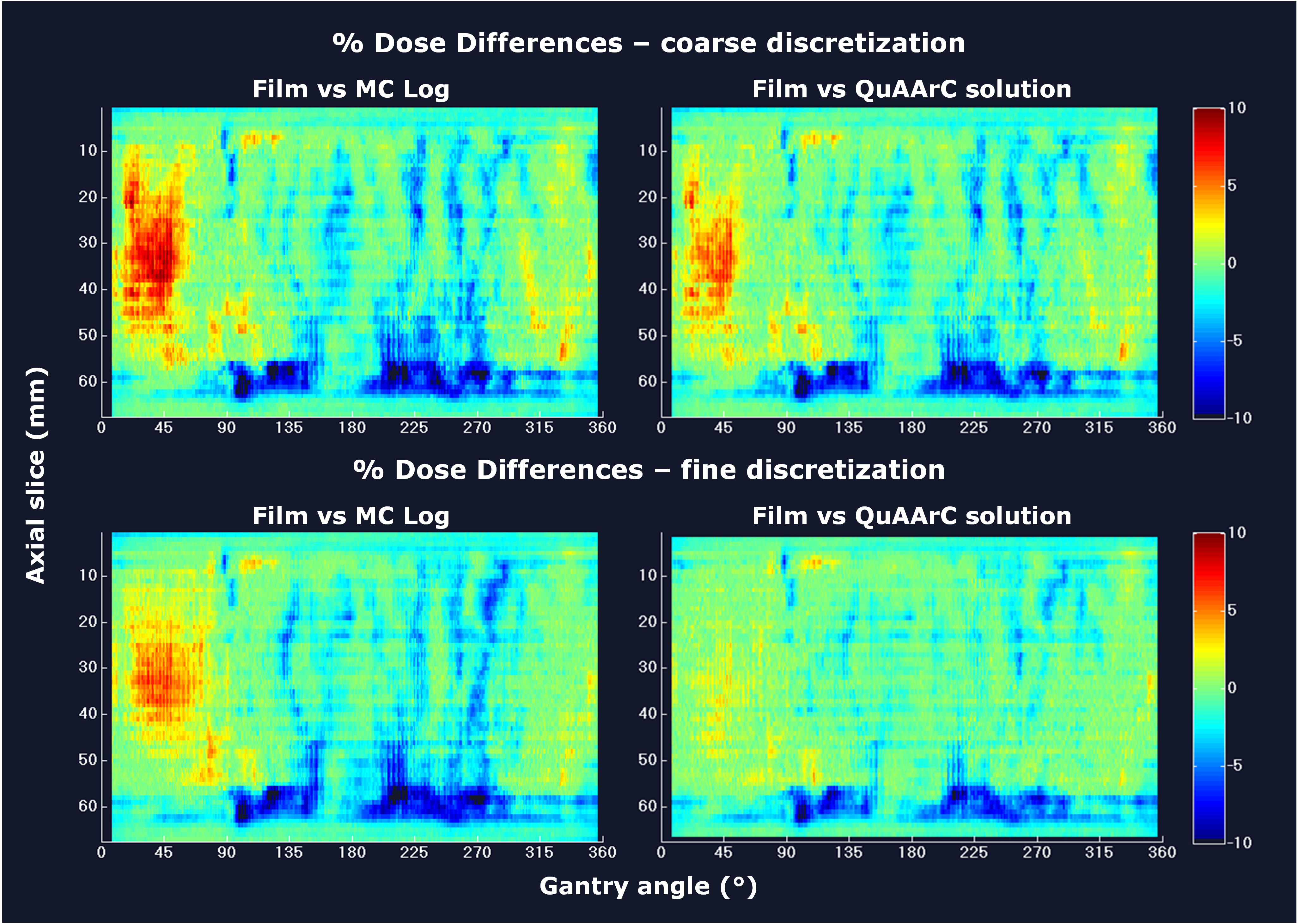

Supplement: S3 Fig — (TIF) [file pone.0166767.s003.tif]

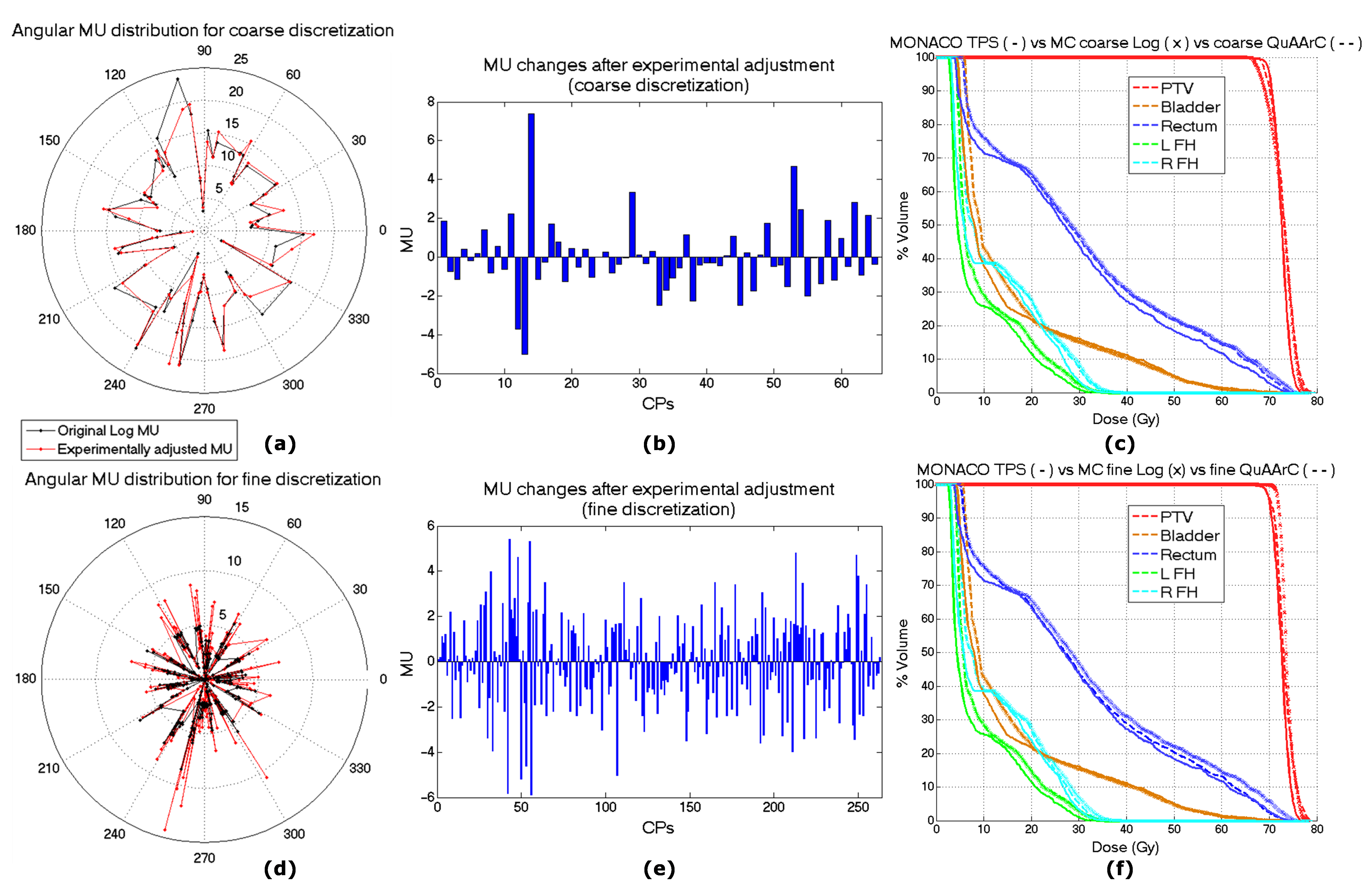

Supplement: S4 Fig — (TIF) [file pone.0166767.s004.tif]

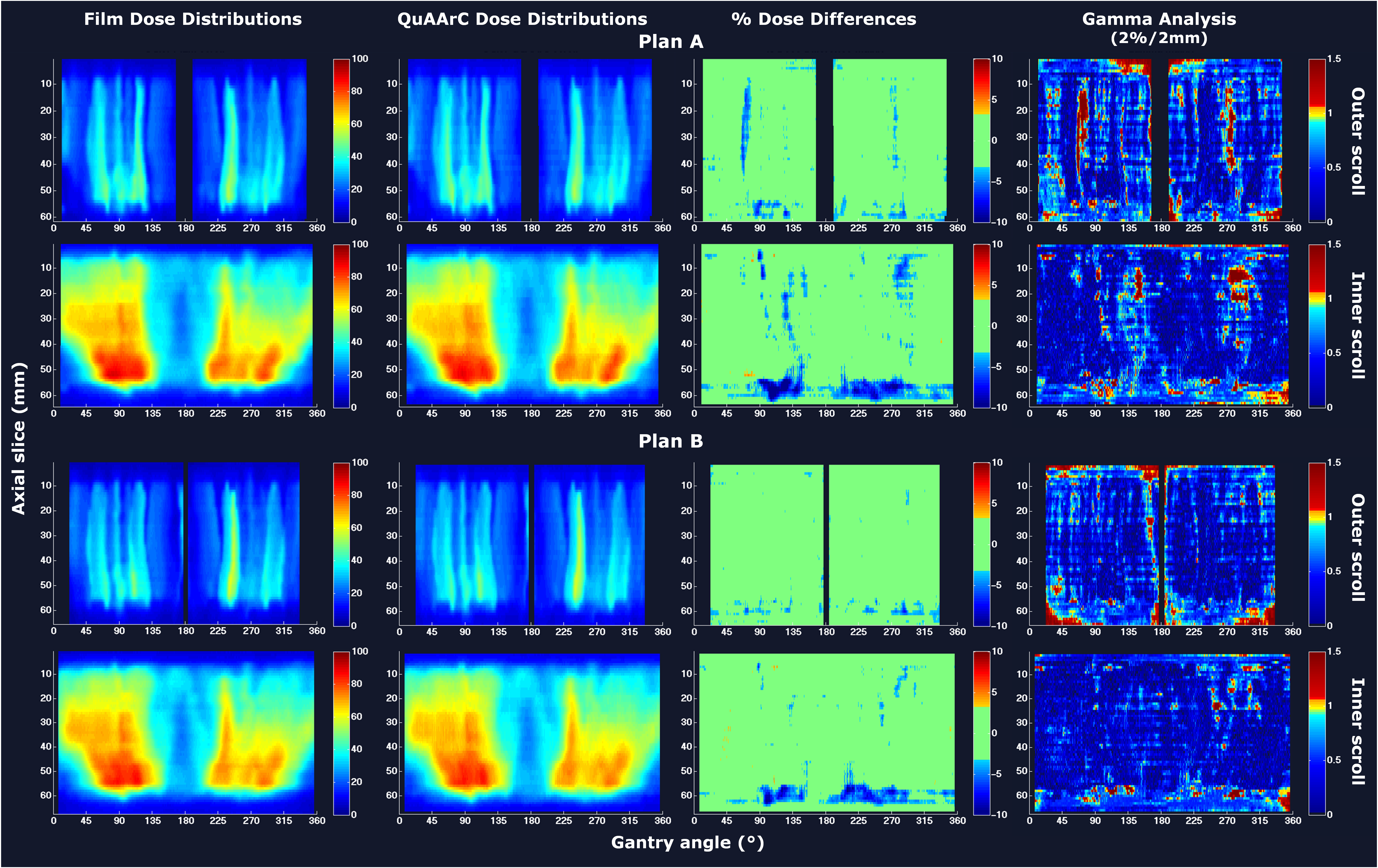

Supplement: S5 Fig — (TIF) [file pone.0166767.s005.tif]

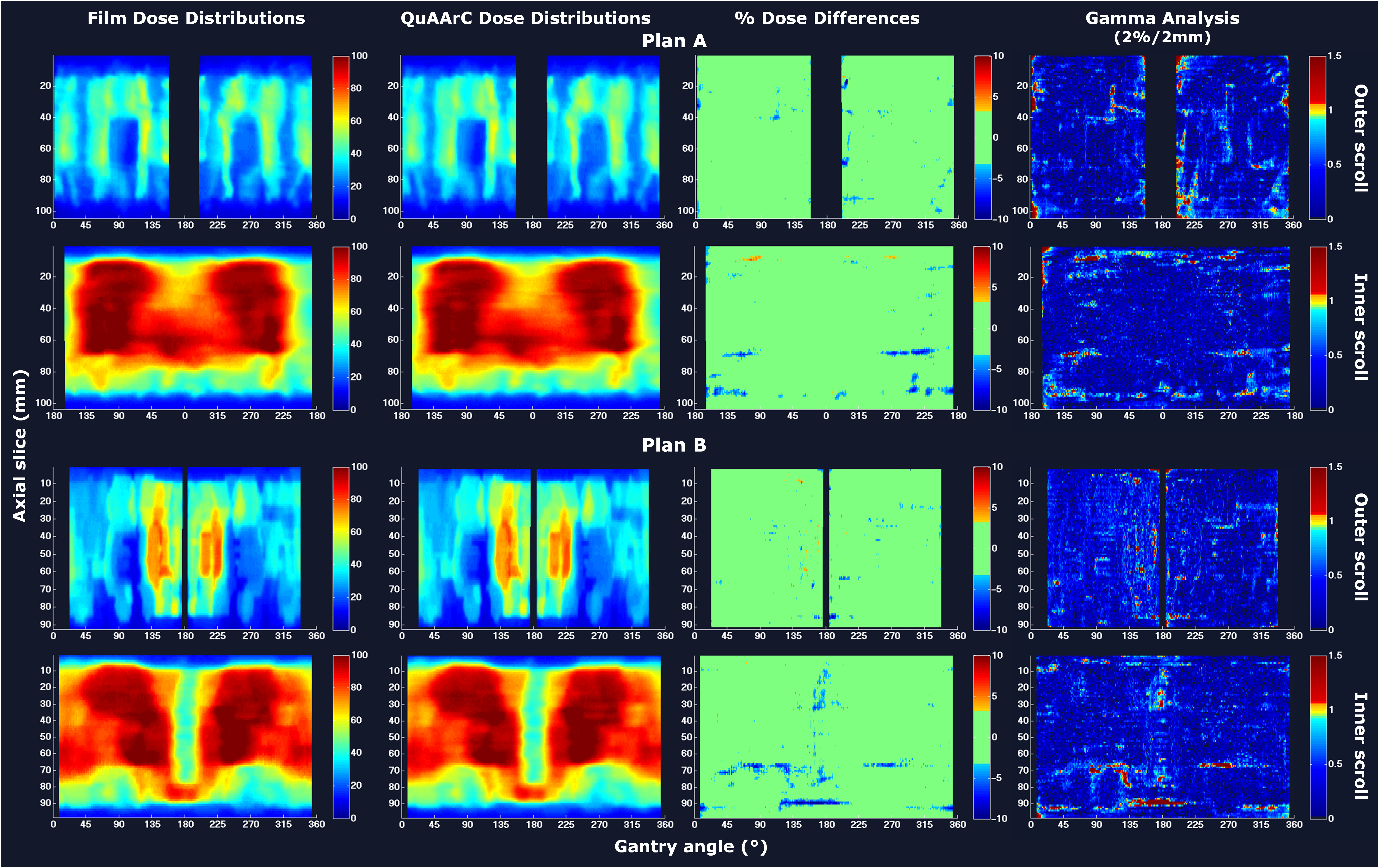

Supplement: S6 Fig — (TIF) [file pone.0166767.s006.tif]

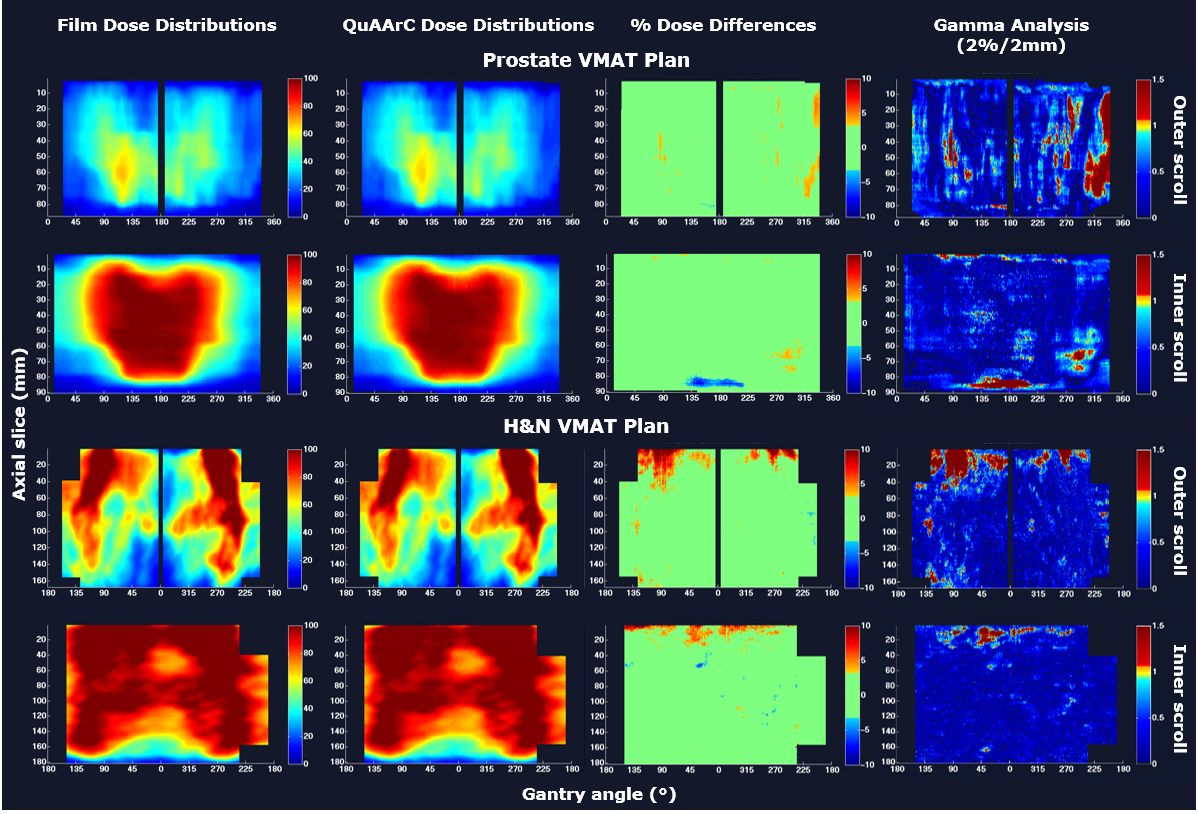

Supplement: S7 Fig — (TIF) [file pone.0166767.s007.tif]

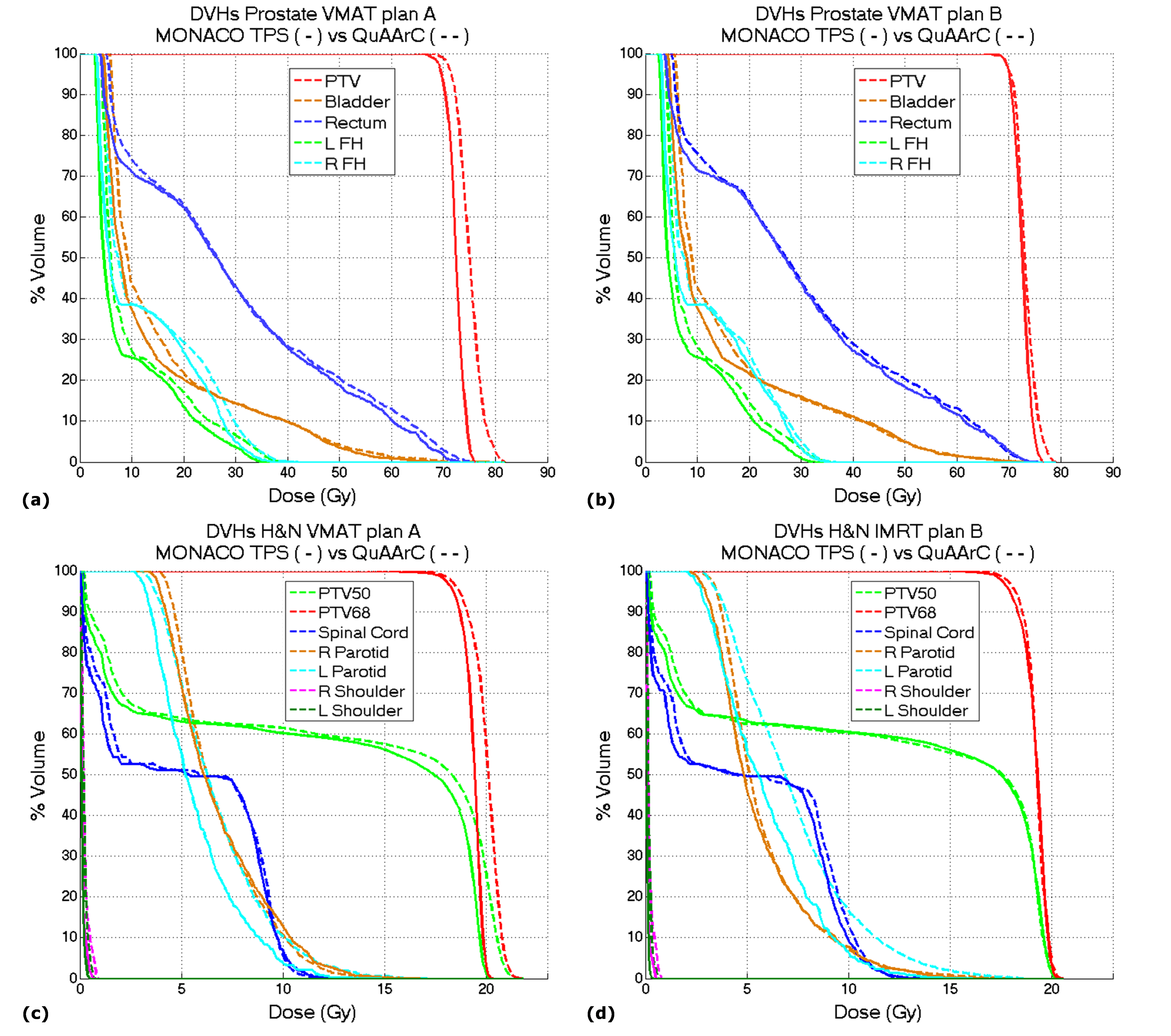

Supplement: S8 Fig — (TIF) [file pone.0166767.s008.tif]

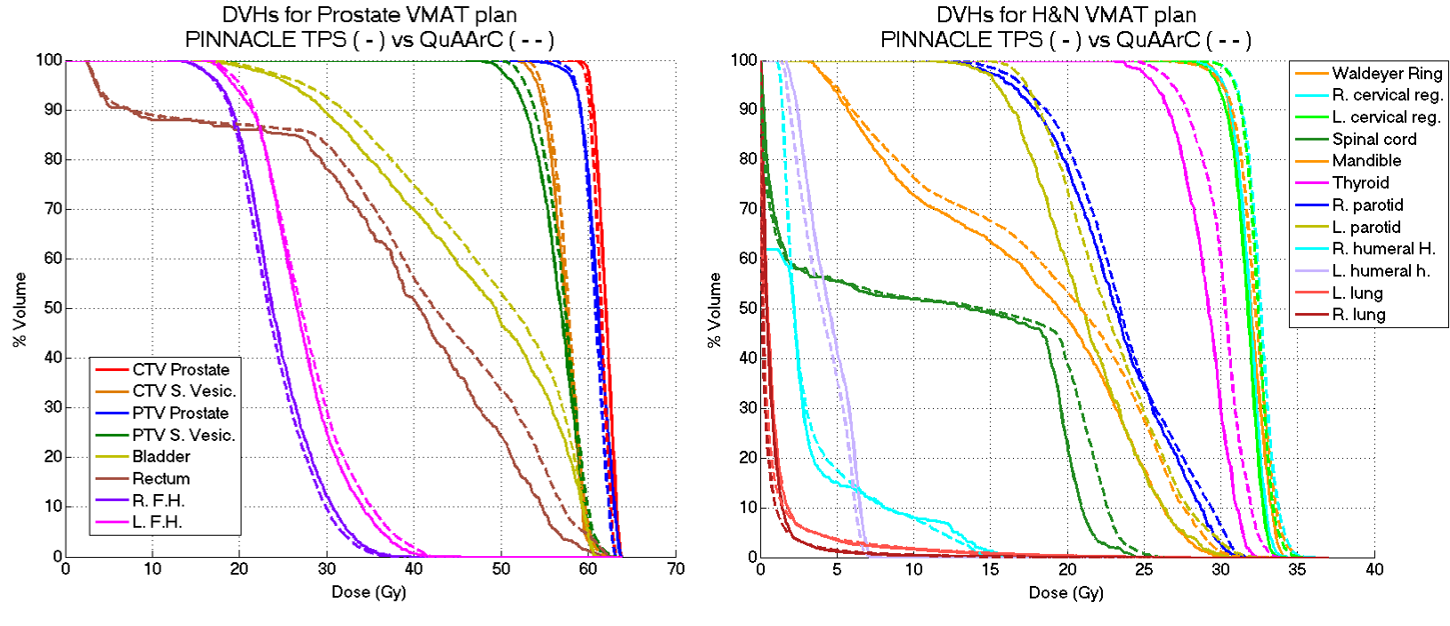

Supplement: S9 Fig — (TIF) [file pone.0166767.s009.tif]
