# Supplementary material for: 3D VMAT Verification Based on Monte Carlo Log File Simulation with Experimental Feedback from Film Dosimetry
Source: PLoS One. 2016 Nov 21;11(11):e0166767. doi: 10.1371/journal.pone.0166767 (PMC5117721; doi:10.1371/journal.pone.0166767)
Supplement: S1 Table — (PPTX) [file pone.0166767.s010.pptx]

## Slide 1
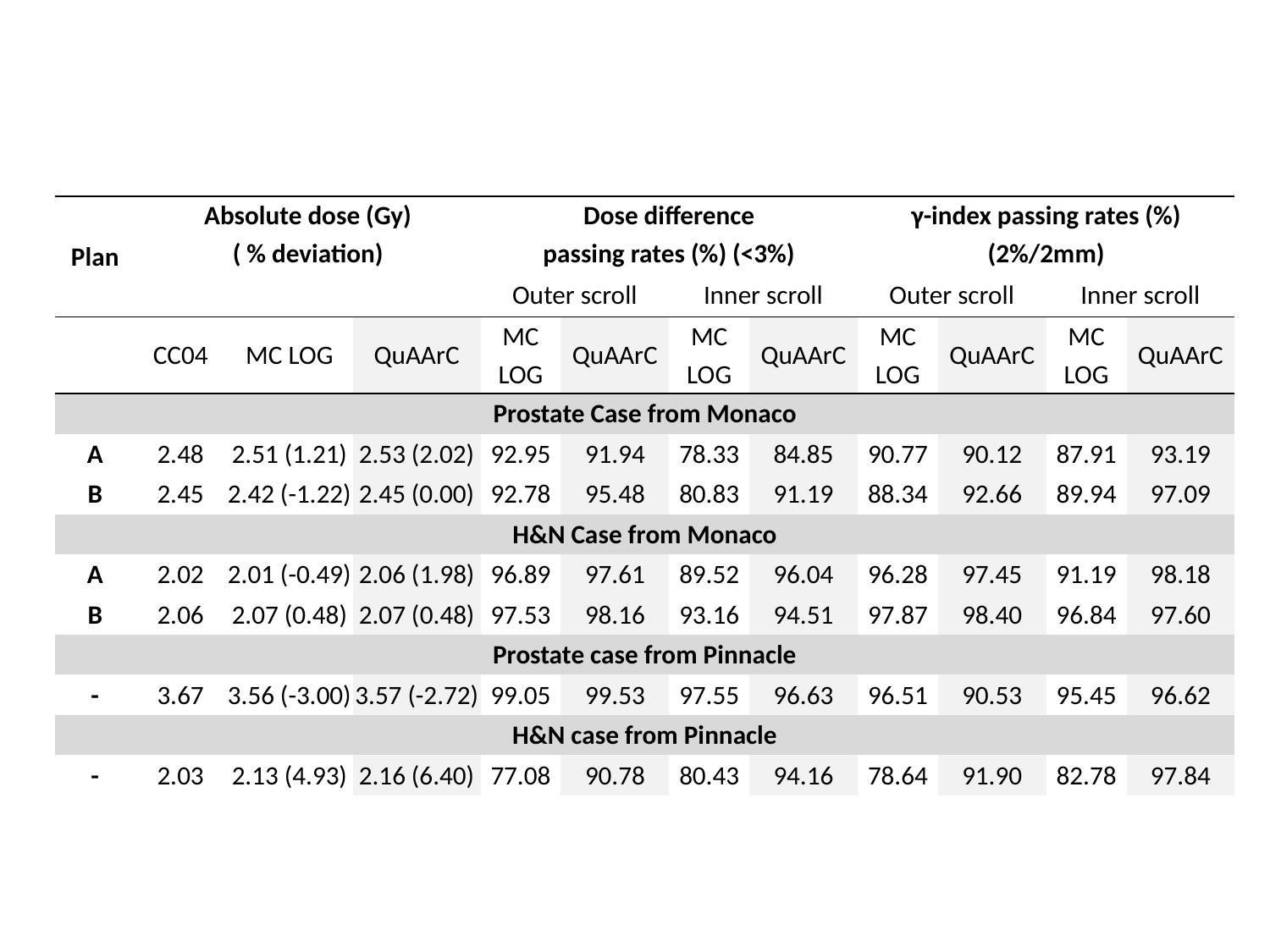

| Plan | Absolute dose (Gy) | | | Dose difference | | | | γ-index passing rates (%) | | | |
| --- | --- | --- | --- | --- | --- | --- | --- | --- | --- | --- | --- |
| | ( % deviation) | | | passing rates (%) (<3%) | | | | (2%/2mm) | | | |
| | | | | Outer scroll | | Inner scroll | | Outer scroll | | Inner scroll | |
| | CC04 | MC LOG | QuAArC | MC | QuAArC | MC | QuAArC | MC | QuAArC | MC | QuAArC |
| | | | | LOG | | LOG | | LOG | | LOG | |
| Prostate Case from Monaco | | | | | | | | | | | |
| A | 2.48 | 2.51 (1.21) | 2.53 (2.02) | 92.95 | 91.94 | 78.33 | 84.85 | 90.77 | 90.12 | 87.91 | 93.19 |
| B | 2.45 | 2.42 (-1.22) | 2.45 (0.00) | 92.78 | 95.48 | 80.83 | 91.19 | 88.34 | 92.66 | 89.94 | 97.09 |
| H&N Case from Monaco | | | | | | | | | | | |
| A | 2.02 | 2.01 (-0.49) | 2.06 (1.98) | 96.89 | 97.61 | 89.52 | 96.04 | 96.28 | 97.45 | 91.19 | 98.18 |
| B | 2.06 | 2.07 (0.48) | 2.07 (0.48) | 97.53 | 98.16 | 93.16 | 94.51 | 97.87 | 98.40 | 96.84 | 97.60 |
| Prostate case from Pinnacle | | | | | | | | | | | |
| - | 3.67 | 3.56 (-3.00) | 3.57 (-2.72) | 99.05 | 99.53 | 97.55 | 96.63 | 96.51 | 90.53 | 95.45 | 96.62 |
| H&N case from Pinnacle | | | | | | | | | | | |
| - | 2.03 | 2.13 (4.93) | 2.16 (6.40) | 77.08 | 90.78 | 80.43 | 94.16 | 78.64 | 91.90 | 82.78 | 97.84 |
